# Letter to the editor RE: Reuling et al., 2018 ‘liver injury in uncomplicated malaria is an overlooked phenomenon: An observational study’

**DOI:** 10.1016/j.ebiom.2021.103377

**Published:** 2021-05-25

**Authors:** Arjen M. Dondorp, Rob W. van der Pluijm, Nicholas P. Day, Nicholas J. White

**Affiliations:** a Mahidol Oxford Tropical Medicine Research Unit (MORU), Faculty of Tropical Medicine Mahidol University, Bangkok, Thailand; b Centre for Tropical Medicine and Global Health, Nuffield Department of Medicine, University of Oxford, Oxford, UK

Reuling et al. report that liver enzyme abnormalities (transient increases in aspartate transaminase (AST) and alanine transaminase (ALT)) [1] are common in experimental *P. falciparum* Controlled Human Malaria Infections (CHMI) in healthy volunteers and uncomplicated falciparum malaria in returning travellers.

This prompted us to review prospectively collected data on these biochemical markers of liver injury from a recently published antimalarial treatment trial in patients with uncomplicated falciparum malaria in Southeast Asia [2]. We confined our analysis to patients treated with conventional artemisinin combination therapies (ACTs) (Table 1).

**Table 1:** Characteristics of patients included in the analysis; all values are median (IQR) or total number (%). DHA-PPQ: dihydroartemisinin-piperaquine; AS-MQ: artesunate-mefloquine; AL: artemether-lumefantrine; AST: Aspartate transaminase; ALT: alanine transaminase; ULN: Upper limit of normal. *Two patients suffered from a grade 3 (severe) ALT and/or AST increase which scored as a grade 3 level at day 7 (2).

	Day 0	Day 3	Day 7	Day 28	Peak concentration during study
Number of subjects	250	250	243	229	250
Age	27·0 (19·0-35·0)				
Gender (Male/total) (%)	215/250 (86·0)	215/250 (86·0)	208/243 (85·6)	197/229 (86·0)	
Temperature	37·5 (36·9–38·1)	36·4 (36·0–36·7)	36·4 (36·0–36·7)	36·3 (36·0–36·7)	
Treatment					
DHA-PPQ (n, % of total)	61 (24·4)	61 (24·4)	59 (24·3)	50 (21·8)	
AS-MQ (n, % of total)	73 (29·2)	73 (29·2)	71 (29·2)	69 (30·1)	
AL (n, % of total)	116 (46·4)	116 (46·4)	113 (46·5)	110 (48·0)	
Parasitaemia at baseline (parasites/microliter)	26,376 (8,672-70,462)				
AST (U/L)	34 (28–42)	30 (24–40)	35 (29–47)	31 (26–39)	44 (34–59)
ALT (U/L)	24 (18–34)	26 (19–36)	34 (24–48)	22 (17–31)	38 (26–55)
Alkaline phosphatase (U/L)	255 (215–361)	253 (204–340)	257 (214–336)	249 (206–334)	289 (236–404)
Bilirubine (total) umol/L	1·0 (0·7–1·5)	0·5 (0·3–0·6)	0·5 (0·4–0·6)	0·5 (0·4–0·7)	1 (0·7–1·5)
**Liver enzyme abnormalities**					
None (< 1·0xULN)	96 (38·4)	117 (46·8)	82 (33·7)	113 (49·3)	
Mild (> 1·0 ≤ 2·5 × ULN)	137 (54·8)	120 (48·0)	139 (57·2)	109 (47·6)	
Moderate (> 2·5 ≤ 5·0xULN)	16 (6·4)	10 (4·0)	18 (7·4)	6 (2·6)	
Severe (> 5·0xULN)	1 (0·4)	3 (1·2)*	4 (1·7)*	1 (0·4)	

Of 250 patients enrolled, 7/250 (2·8%), had at least one significant increase (> 5·0x ULN) in AST and/or ALT (Fuji DRI-CHEM 4000I) during the study. Most of these increases were observed first at day 3. This compared to 16/187 (8·8%) in experimentally infected individuals in the CHMI model (p = 0.008). In our study, median (range; IQR) peak plasma AST was 44 U/L (21-505; 34–59) and ALT 38 U/L (9-404; 26–55), compared to 52 U/L (22-723; 43–85) and 69 U/L (13–870; 46–98) in the CHMI model despite 100 to 1000 times lower parasite densities.

In a recent large randomised trial, comparing 4 different ACTs which recruited 4710 children with uncomplicated falciparum malaria, the incidence of a > 5-fold increases in ALT or AST after antimalarial treatment was below 2·0% [3]. These incidence rates are well below the reported increases in transaminases in returning travellers. The high peak levels of ALT and AST and high incidence of severe transaminase abnormalities in the CHMI model at very low parasite densities deserve further attention, since the laboratory-adapted strains used in this model could have an altered pathogenicity. Other elements of the model might also contribute to the observed ‘transaminitis’. Awareness of potential model related adverse events is important. ‘False flags’ could discredit novel antimalarials that would not be hepatotoxic in the treatment of uncomplicated malaria in the field. In an era of increasing antimalarial drug resistance problems, any unnecessary delay in the development of novel antimalarial drugs should be avoided.

## Declaration of Competing Interest

The authors declare no competing interests.

## Appendix Supplementary materials

Image, application 1
