# Supplementary material for: Letter to the editor RE: Reuling et al., 2018 ‘liver injury in uncomplicated malaria is an overlooked phenomenon: An observational study’
Source: eBioMedicine. 2021 May 25;68:103377. doi: 10.1016/j.ebiom.2021.103377 (PMC8170069; doi:10.1016/j.ebiom.2021.103377)
Supplement: Supplementary file 1 [file mmc1.docx]

| Table 1. | Day 0 | Day 3 | Day 7 | Day 28 | Peak level during study |
| --- | --- | --- | --- | --- | --- |
| Number of subjects | 250 | 250 | 243 | 229 | 250 |
| Age | 27·0  (19·0-35·0) | | | |  |
| Gender (Male/total) (%) | 215/250 (86·0) | 215/250 (86·0) | 208/243 (85·6) | 197/229 (86·0) |  |
| Temperature | 37·5  (36·9-38·1) | 36·4  (36·0-36·7) | 36·4  (36·0-36·7) | 36·3  (36·0-36·7) |  |
| Treatment |  | | | |  |
| DHA-PPQ (n, % of total) | 61 (24·4) | 61 (24·4) | 59 (24·3) | 50 (21·8) |  |
| AS-MQ (n, % of total) | 73 (29·2) | 73 (29·2) | 71 (29·2) | 69 (30·1) |  |
| AL (n, % of total) | 116 (46·4) | 116 (46·4) | 113 (46·5) | 110 (48·0) |  |
| Parasitaemia at baseline (parasites/microliter) | 26,376  (8,672-70,462) |  |  |  |  |
| AST (U/L) | 34 (28-42) | 30 (24-40) | 35 (29-47) | 31 (26-39) | 44 (34-59) |
| ALT (U/L) | 24 (18-34) | 26 (19-36) | 34 (24-48) | 22 (17-31) | 38 (26-55) |
| Alkaline phosphatase (U/L) | 255 (215-361) | 253 (204-340) | 257 (214-336) | 249 (206-334) | 289 (236-404) |
| Bilirubine (total) umol/L | 1·0 (0·7-1·5) | 0·5 (0·3-0·6) | 0·5 (0·4-0·6) | 0·5 (0·4-0·7) | 1 (0·7-1·5) |
| **Liver enzyme abnormalities** | | | | | |
| None (<1·0xULN) | 96 (38·4) | 117 (46·8) | 82 (33·7) | 113 (49·3) |  |
| Mild (>1·0 ≤ 2·5×ULN) | 137 (54·8) | 120 (48·0) | 139 (57·2) | 109 (47·6) |  |
| Moderate (>2·5 ≤ 5·0xULN) | 16 (6·4) | 10 (4·0) | 18 (7·4) | 6 (2·6) |  |
| Severe (>5·0xULN) | 1 (0·4) | 3 (1·2)* | 4 (1·7)* | 1 (0·4) |  |

Table 1. Characteristics of patients included in the analysis; all values are median (IQR) or total number (%). DHA-PPQ: dihydroartemisinin-piperaquine; AS-MQ: artesunate-mefloquine; AL: artemether-lumefantrine; AST: Aspartate transaminase; ALT: alanine transaminase; ULN: Upper limit of normal. *Two patients suffered from a grade 3 (severe) ALT and/or AST increase which scored as a grade 3 level at day 7.
